# COVID-19 Vaccine Acceptance Level in Ethiopia: A Systematic Review and Meta-Analysis

**DOI:** 10.1155/2022/2313367

**Published:** 2022-08-25

**Authors:** Addisu Tadesse Sahile, Girma Demissie Gizaw, Tennyson Mgutshini, Zewdu Minwuyelet Gebremariam, Getabalew Endazenaw Bekele

**Affiliations:** ^1^Department of Public Health, Unity University, Addis Ababa, Ethiopia; ^2^Department of Public Health Emergency Management, Ethiopian Public Health Institute, Addis Ababa, Ethiopia; ^3^Department of Public Health, University of South Africa, Pretoria, South Africa; ^4^Department of Biomedical Science, Kotobe Metropolitan University, Addis Ababa, Ethiopia; ^5^Department of Public Health, Yekatit 12 Hospital Medical College, Addis Ababa, Ethiopia

## Abstract

**Background:**

The coronavirus disease 2019 pandemic has had a devastating impact on the everyday lives of the world's population and to this end, the development of curative vaccines was upheld as a welcome panacea. Despite the undeniable negative impact of the disease on human beings, lower than expected proportions of people have taken up the vaccines, particularly in the developing non-Western world. Ethiopia represents an interesting case example, of a nation where COVID-19 vaccine acceptance levels have not been well investigated and a need exists to assess the overall level of vaccine acceptance.

**Methods:**

A systematic multidatabase search for relevant articles was carried out across Google Scholar, Web of Science, Science Direct, Hinari, EMBASE, Boolean operator, and PubMed. Two reviewers independently selected, reviewed, screened, and extracted data by using a Microsoft Excel spreadsheet. The Joanna Briggs Institute prevalence critical appraisal tools and the modified NewcastleOttawa Scale (NOS) were used to assess the quality of evidence. All studies conducted in Ethiopia, reporting vaccine acceptance rates were incorporated. The extracted data were imported into the comprehensive meta-analysis version 3.0 for further analysis. Heterogeneity was confirmed using Higgins's method, and publication bias was checked by using Beggs and Eggers tests. A random-effects meta-analysis model with a 95% confidence interval was computed to estimate the pooled prevalence. Furthermore, subgroup analysis based on the study area and sample size was done. *Results and Conclusion*. After reviewing 67 sources, 18 articles fulfilled the inclusion criteria and were included in the meta-analysis. The pooled prevalence of COVID-19 vaccine acceptance in Ethiopia was 57.8% (95% CI: 47.2%–67.8%). The level of COVID-19 vaccine acceptance in Ethiopia was at a lower rate than necessary to achieve herd immunity. The highest level of vaccine acceptance rate was reported via online or telephone surveys followed by the southern region of Ethiopia. The lowest vaccine acceptance patterns were reported in Addis Ababa.

## 1. Introduction

The coronavirus (COVID)-19 disease is caused by a highly contagious acute respiratory syndrome coronavirus 2 (SARS-CoV-2) and for several months after its emergence, no proven vaccine was available. Globally, the fatality rate of COVID-19 infection was estimated at 0.5% to 1% [1]. Its negative impact reached the everyday lives of all human beings globally [2–5] to the extent of disrupting the normal economic and social activities of the world's population [6–10]. In response, global communities took different measures to contain its spread, and these included lockdowns and border closures [11–13].

There has been an implementation of various public health measures that included hand hygiene, lockdowns, and social distancing in most parts of the world. Even so, the overall impact of these COVID-19 disease prevention measures has varied from one set to the other [14]. Medically, vaccines have been separated from other preventative measures on the basis of their superior prevention and disease control profiles [15, 16]. Even so, their uptake remains an issue of contention.

Barriers to vaccine acceptance are complex; moreover, they are context-specific and fluctuate across the place, time, and vaccine type [17]. However, there has been a continuous distribution of COVID-19 vaccines across the world population including Ethiopia. That said, hesitation against vaccines represents the single most notable obstacle to having adequate coverage across various populations. Vaccine hesitation was identified by the World Health Organization as one of the top global health threats as of the year 2019 [18].

The corpus of evidence related to COVID-19 vaccine acceptance suggests that a considerable portion of people are opposed to the vaccine. A global report on COVID-19 vaccine acceptance reported the acceptance rate to be under 67% [19].

Notably, most reports in Ethiopia show lower vaccine coverage. Additionally, the lack of specified investigation of the above strongly points to an acknowledgment that, the overall level of COVID-19 vaccine acceptance in Ethiopia represents a poorly investigated phenomenon. Guided by this, this meta-analysis offers an assessment of the overall level of vaccine acceptance in Ethiopia.

### 1.1. Question

What is the overall level of COVID-19 vaccine acceptance in Ethiopia?

## 2. Methods

### 2.1. Reporting

The preferred Reporting Items for Systematic Reviews and Meta-Analyses (PRISMA) guideline [20] was used as a reporting framework within this meta-analysis (additional file 1 S1).

### 2.2. Searching Strategies

The PRISMA systematic review protocol was followed as a reporting guideline and eligible studies for the analysis were selected in terms of abstracts, titles, and then for full articles on the basis of the inclusion criteria. EMBASE, PubMed, Hinari, Google Scholar, Web of Science, Science Direct, and African Journals Online were systematically searched to identify articles that included Medical Subject Headings and free-text languages. These databases were searched by using both controlled and free-text languages. In terms of free-text searches, the keywords included the following combination of terms: (Willingness, OR COVID-19 Vaccine OR Acceptance) AND Ethiopia. The controlled searches included the following Medical Subject Headings (MeSH) terms: “COVID-19 Vaccine Acceptance” and “Ethiopia” as recommended for each database. Search terms were used individually and in combination using “AND” and “OR” Boolean operators. The search was guided by PICO, a population that was intended to take the vaccine.

### 2.3. Inclusion and Exclusion Criteria

The following types of studies from 2019 to 2022 were included; study populations comprised any age group, study outcome was “willingness or intention to take vaccine or vaccine acceptance,” study design is cross-sectional and studies written in English were included. However, in this systematic review and meta-analysis; qualitative studies and data on those who took the vaccine were excluded.

### 2.4. Outcome of Interest: PICO

The population of the study was any age group and the outcome of interest was vaccine acceptance which thus was reported as “Are you willing to take COVID-19 Vaccine if available to you?” and measured as yes and no or willing versus unwilling. The level of vaccine acceptance was presented as a frequency and percentage.

### 2.5. Screening and Data Extraction

Screening for titles and abstracts against the inclusion was conducted by the two reviewers (SAT and GGD). Furthermore, an independent assessment was made for the full-text articles based on the predetermined inclusion criteria. Inconsistencies across the reviewers were dealt with via a discussion and consensus-seeking engagements involving all the investigators. Data extraction was made by three authors (TM, GZM, and BGE) independently from a random sample of 20% of the papers to check consistency and cross variation.

### 2.6. Study Quality Assessment

A structured data abstraction form was constructed in Microsoft Excel. Attention was given to clarity of data, objective, study design, population, sample size, and proportion of vaccine acceptance (Table 1). The modified version of the NewcastleOttawa Scale for the cross-sectional study [39] was used for the methodological qualities of each article. Additionally, studies were critically appraised with the Joanna Briggs Institute prevalence critical appraisal tool [40].

### 2.7. Data Synthesis and Statistical Analysis

Data were extracted using a Microsoft Excel spreadsheet and imported to comprehensive meta-analysis version3.0 software for further analysis. The pooled effect size with a 95% confidence interval of national COVID-19 vaccine acceptance; a rate that was determined using a weighted inverse variance random-effects model. The *I*^2^ statistic; 25, 50, and 75% representing a low, moderate, and high heterogeneity consecutively assessed the heterogeneity across the studies [41], whereas the publication bias was evaluated by funnel plot and Eggers and Beggs test [42].

## 3. Result

### 3.1. Selection of the Studies

**Figure 1:**
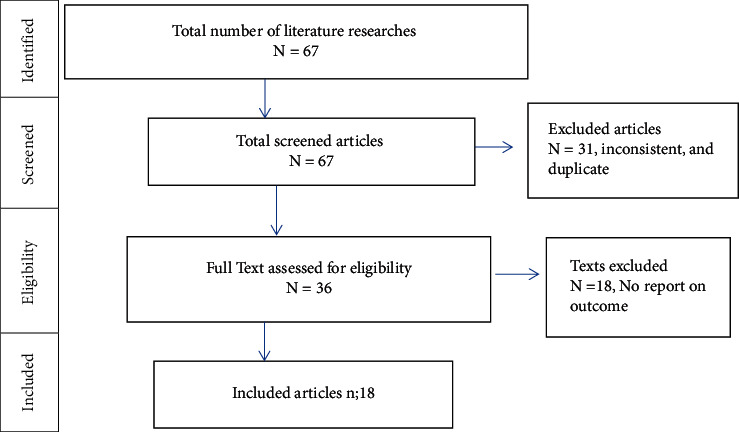
PRISMA flow chart for showing the screening and selection process of studies.

A comprehensive literature search of the databases yielded a total of 67 published articles, of which 20 articles were retrieved from Google Scholar, 13 articles from PubMed, 12 articles from African Journals online, 7 articles from Hinari, and 15 articles from EMBASE, Web of Science, and Science Direct. Thirty-one articles were excluded for duplication and scope. The other 18 articles were excluded for failing to offer reports on the outcome. A total of 18 full-text articles that fulfilled the eligibility criteria with a total sample size of 10873 were included in the final analysis for the systematic review and meta-analysis (Figure 1).

### 3.2. Characteristics of the Included Studies

Pertinent data relating to “year of publication,” authors, the outcome, and sample sizes with other main findings from the selected articles were extracted and presented in Table 1. All articles were cross-sectional and conducted in Ethiopia. The studies were distributed in southern region [21, 27–29, 31], Addis Ababa [22–24, 36], Amhara region [25, 26, 30, 33, 37], and online or telephone. [32, 34, 35, 38]. The sample size of the selected studies ranged from 301 to 2178 (Table 1).

### 3.3. Subgroup Analysis

According to the subgroup analysis report, the highest level of vaccine acceptance (68.7%; 95% CI: 34.1%–90.3%) was reported in online or telephone surveys whilst the lowest level of vaccine acceptance (51.8%; 95% CI: 33.3%–69.8%) was reported in Addis Ababa. Regarding the sample size, the highest level of vaccine acceptance (74.0%; 95% CI: 23.5%–96.4%) was reported in studies with a sample size of larger than 800 (Table 2).

### 3.4. COVID-19 Vaccine Acceptance

**Figure 2:**
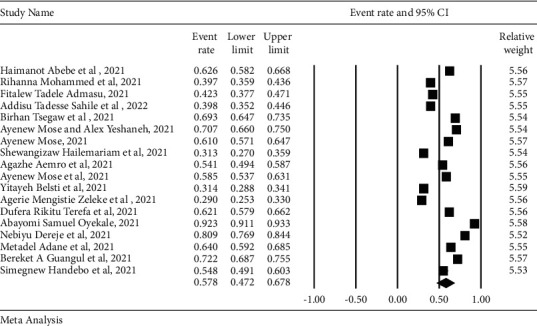
Forest plot for the pooled prevalence of COVID-19 vaccine acceptance.

In this systematic review and meta-analysis, the pooled estimate of the COVID-19 vaccine acceptance rate was illustrated via a forest plot. The pooled prevalence of vaccine acceptance in Ethiopia was 57.8% (95% CI: 47.2%–67.8%) (Figure 2).

### 3.5. Assessment of Publication Bias

**Figure 3:**
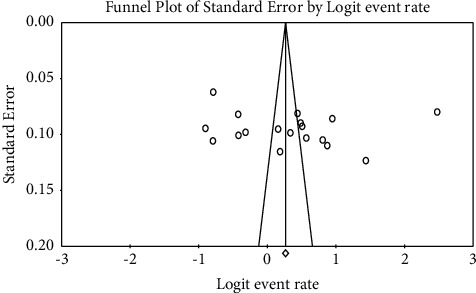
Funnel plot of the included studies.

A symmetrical funnel plot was observed. Begg's and Egger's tests showed the absence of significant publication bias at a *p* value of >0.05 (Figure 3).

### 3.6. Heterogeneity

For the identification of the possible causes of variation across different studies, meta-regression analysis was conducted using sample size and study area. The result showed that there was no significant heterogeneity across the studies (*p* > 0.05) (Table 3).

## 4. Discussion

The current systematic review and meta-analysis provided critical evidence on the level of COVID-19 vaccine acceptance in Ethiopia. This study found the overall level of vaccine acceptance in Ethiopia to be at 58.7%. This was consistent with the findings of 48.93% in Africa [43], 58.5% in low and middle income countries [44], 20.0% to 58.2% in Nigeria [45], 48% among adults in Saudi Arabia [46], 67% among adults in Kuwait [47], 62.6% in Jordan [48], 63% in Hong Kong [49], 65.75% in Japan [50], 53% in Pakistan [51], 61.7% in Iraq [52], 65.6% in Qatar [53], and 51.6% in Turkey [54]. The finding from this study was also consistent with the results from 64% in the UK (56), 63.5% in Kenya [55], and 50.2% in Nigeria [56].

A higher level of vaccine acceptance was reported in different countries. These included Malawi with an overall prevalence of 82.7% [57], 71.4% in Mozambique [58], 84.9% in Rwanda [59], 71.0% in Côte d'Ivoire [60], 75.3% in China [61], 74.65% among adults in Bangladesh [62], 85% in Israel [63], 91.3% of adults in China [15], 94.3% in Malaysia [64], and 91.5% in Italy [65]. This variation might be due to variation in the availability of vaccine type and population characteristics.

By contrast, the level of vaccine acceptance in the current study was higher than the findings of 34% in Liberia [66], 21.4% in Lebanon [67], and 27.7% in the Democratic Republic of Congo [68]. Such variation might be due to variations in sample size and level of awareness among the study participants.

The vaccine acceptance level was higher in different countries including 78% in Scotland [69], 78.5% in Greece [70], 75% in Portugal [71], 77.65% in France [72], 68.5% in the United States [73], 80% in Canada [74], 90.1% in South Africa [75], and 80.9% in Uganda [76]. This difference might be due to population characteristics and the availability of vaccine options.

## 5. Conclusion

The level of COVID-19 vaccine acceptance in Ethiopia was at a lower rate than necessary to achieve herd immunity. The highest level of vaccine acceptance was reported in online or telephone surveys followed by the Southern region of Ethiopia whereas a lower level of vaccine acceptance was reported in Addis Ababa. With regards to the sample size, the highest level of vaccine acceptance was reported in studies with a sample size larger than 800. Concerned bodies in Ethiopia including the government should work on scaling up the vaccine coverage for the Ethiopian people.

## Figures and Tables

**Table 1:** Characteristics of included studies, their area, sample size, and outcome.

Author	Year	Region	Sample size	Prevalence (%)
Abebe et al. [21]	2021	Southern, (Gurage)	492	62.60
Mohammed et al. [22]	2021	Addis Ababa	614	39.70
Tadele Admasu [23]	2021	Addis Ababa	422	42.30
Sahile et al. [24]	2022	Addis Ababa	407	39.80
Tsegaw et al. [25]	2021	Debre Berhan	423	69.30
Handebo et al. [26]	2021	Gondar	301	54.80
Mose and Yeshaneh [27]	2021	Southwest (Wolkite)	396	70.70
Mose [28]	2021	Southern	630	61.0
Hailemariam et al. [29]	2021	Southwest	412	31.30
Aemro et al. [30]	2021	Northwest	440	54.10
Mose et al. [31]	2021	Southwest	420	58.80
Belsti et al. [32]	2021	Online	1184	31.40
Zeleke and Bayeh [33]	2021	Northwest	538	29.00
Rikitu Terefa et al. [34]	2021	Online	522	62.10
Oyekale [35]	2021	Telephone	2178	92.30
Dereje et al. [36]	2021	Addis Ababa	422	80.90
Adane et al. [37]	2021	Northeast (Dessie)	404	64.0
Bereket et al. [38]	2021	Online	668	72.20

**Table 2:** Level of COVID-19 vaccine acceptance by study area and sample size.

Variables	Characteristics	Included studies	Sample size	Prevalence (95% CI)
Area	Addis Ababa	4	1865	51.8% (33.3%–69.8%)
	Amhara	5	2106	54.2% (39.4%–68.3%)
	Southern	5	2350	57.0% (44.4%–68.7%)
	Other (online/telephone)	4	4552	68.7% (34.1%–90.3%)

Sample size	<400	2	697	63.5% (59.8%–67.0%)
	400–800	13	6814	55.2% (46.7%–63.4%)
	>800	3	3362	74.0% (23.5%–96.4%)

**Table 3:** Sources of heterogeneity across the studies.

Source of heterogeneity	Coefficient	Standard error	*t * ^2^ (%)	*p* value
Study area	0.0731	0.4951	99.10	0.8827
Sample size	0.529	0.5709	99.08	0.3535

## Data Availability

All the data are contained within the article.
